# Practical Notes and Formulæ

**Published:** 1887-08

**Authors:** 


### PRACTICAL NOTES AND FORMULÆ.

**Hematemesis.**—

℞. Acidi gallici . . . . . . . . . . . . . . . . . . . . . . . . . . . . . . . . . gr. xv,
Aq. dest . . . . . . . . . . . . . . . . . . . . . . . . . . . . . . . . . . . . ℥ jss.

M. To be taken immediately, and repeated, if necessary, in acute hematemesis.

℞. Acidi gallici . . . . . . . . . . . . . . . . . . . . . . . . . . . . . . . . gr. xx,

Acid. sulphurici arom . . . . . . . . . . . . . . . . . . . . . . . . ♏ xv,

Tinct. cinnamomi . . . . . . . . . . . . . . . . . . . . . . . . . . . . ʒ jss,

Aq. dest . . . . . . . . . . . . . . . . . . . . . . . . . . . . . . . . . . . ℥ ij.

M. The draught, every four hours, in acute hematemesis.

℞. Ext. ergotæ liq . . . . . . . . . . . . . . . . . . . . . . . . . . . . . . . ʒ iij,

Aq. cinnamomi ad . . . . . . . . . . . . . . . . . . . . . . . . . . . . ℥ vj.

M. Two tablespoonsful every two or three hours, in acute hemametesis.

**Pruritus Vulvæ**—Dr. Barnes, in *Medical World*, says: "In the April number of the *Medical World*, p. 119, Dr. B. F. Records asks for a remedy for this distressing disease. I cured an unmarried lady, aged twenty eight years, with the following application:

℞. Ung. hydrag, nitrat . . . . . . . . . . . . . . . . . . . . . . . . . . . . ʒ ij,

Acidi carbolici . . . . . . . . . . . . . . . . . . . . . . . . . . . . . . . . gtt. x,

Vaseline . . . . . . . . . . . . . . . . . . . . . . . . . . . . . . . . . . . . . ʒ vij.

S. Rub in well night and morning, and at any time when there is itching.

It must be remembered that the trouble is often caused by some disease of the internal genital organs, and that this must be first cured before relief can be obtained The above named case, a beautifully formed little lady of 90 pounds weight, had anteflexion and prolapsus of the uterus, of which I will write later. I cured a gentleman sixty-five years old of pruritus of the scrotum, etc. of twenty-five years standing, and other cases with the above.

**Diarrhœa of Children.**—With green stools:

℞. Hydrarg. chlor. mite . . . . . . . . . . . . . . . . . . . . . . . . . . gr. ¼,

Pulv. rad. rhei . . . . . . . . . . . . . . . . . . . . . . . . . . . . . . . gr. ¾,

Conch. praep . . . . . . . . . . . . . . . . . . . . . . . . . . . . . . . . . . gr. ivss.

M. Make into 8 powders. Sig.—One powder three or four times daily.

**For Earache**—a gentle stream of water as hot as can be borne, directed into the ear from a fountain syringe, is better than poultices or anodynes.—*Good Health.*

**For Cholera Infantum.**—The following is a slight modification of Ungar's prescription:

℞. Thymol....................................gr. iv,

Brandy or whisky...........................ʒ i,

Syrup of ginger.............................ʒ ss,

Water sufficient to make .........  ...........ʒ iv.

M. Dose from 1 to 2 tablespoonsful, according to the age of infant, repeated as often as necessary.

**Arsenic and Lithia for Diabetes.**—At a meeting of the Paris Societe de Theraputique, February 23, Dr. Martineau recommended the following treatment, with which, he said, he had cured sixty-seven out of seventy patients suffering from arthritic diabetes:

℞. Carbonate of lithium.........................gr. iij,
Arseniate sodium..............................gr. 1-10,
Carbonic acid water............  .............pt. ij.

Effect the solution under pressure. The effervescing liquid is to be drunk at meals, mixed with claret, and the foregoing dose is to last for at least three meals of the day, customary in Paris. No change of diet is necssary. Dr. Martineau's fellow members—Dr. Dujardin-Beaumetz among them—were somewhat skeptical about the value of the treatment, but it is so simple and easy that it can be given a trial when the patient is not dangerously ill.—*Therapeutic Gazette.*

**Delirium of Typhoid Fever.**—Prof. N. S Davis, Chicago:

℞. Strychnins..................................gr. j,

Acidi Nitrici.......................  ....ʒ j,

Tr. Opii......................................ʒ iv,

Aquæ........................................℥ iijss.

M. Sig.—A teaspoonful in sweetened water every two, three, four or six hours, according to the urgency of the symptoms.—*Illustrated Medical Journal.*

**Acute Tonsilitis.**—Dr. O. S. Armstrong, Detroit:

℞. Fl. ext. guaiac................................ʒ ivss,

Syr. simp., q. s...............................℥ iv,

M. Sig.—Teaspoonful every two hours, or ½ teaspoonful every hour in early stages.—*Ib.*

**Acute Rheumatism.**—Prof. J. E. Clark, Detroit:

℞. Acid salicylic................................ʒ ivss,

Sodi bicarb...................................ʒ iij,

Aquæ........................................q. s.

Misce et adde:
Vini colchi....................................℥ iss,
Elix. sim, q. s.................................℥ iv.

Sig.—Two teaspoonfuls every two hours.—*Ib.*

**Intestinal Worms.**—Prof. J. Lewis Smith, New York:

℞. Fl. ext. spigeliæ et sennæ.....................℥ i,

Santonini ..............................gr. viij,

M. Sig.—Teaspoonful to a child of five.—*Ib.*

**Pruritus Vulvæ.**—Prof. Wm. Goodell, Philadelphia:

℞. Hydrarg. chlorid. corros........................gr. i,
Pulv. Aluminis...........................gr. xx,
Amyli.... ...........................ʒ iss,
Aquæ.................................℥ ii.

M. Sig.—Apply locally.

**Cholera Infantum.**—Prof. N. S. Davis, Chicago:

℞. Sodii bicarbonat...........................ʒ i,

Morph. sulphat.............................gr. i,

Aquæ.................... ................℥ ij.

M. Sig.—From 6 to 15 minims, according to the age of the patieut, immediately after each paroxysm of vomiting.—*Ib.*

**Chronic Eczema.**—Prof. Da Costa, Philadelphia:

℞. Ung.Hydrarg Oxidi Rubri ......................ʒ ij,

Unguent. sulphuris..........................ʒ ij,

Acid carbolic..............................gr. iij,

Ungent. simplicis...........................℥ ss.

M. Sig.—Apply to the affected part.—*Ib.*

**Infantile Convulsions.**—Prof. A. Jacobi, New York: First give a purgative dose of calomel, and follow in a few hours by—

℞. Chloral Hydrat ..................... ......gr. iv,
Potassi Bromidi..................... ...gr. viij,
Aquæ..............................
Syrupi.............................aa ʒ i.

M. Sig.—Dose for a child two years old.—*Ib.*

**Thread-worms in Children.**—Dr. Martin:

℞. Tincturæ rhei............................*m* iii,
Magnesii carbonatis.......................gr. iii,
Tincturæ zingiberis.......................*m* i,
Aquæ................................f ʒ i.

M. To be taken twice or three times daily.

**Myrrh as a Preventive of Infectious Maladies.**—Dr. W. Femple recommends the keeping of a fragment of myrrh in the mouth during exposure in a dangerously infected locality, and felicitates himself on having observed this precaution in various epidemics. He considers myrrh as an excellent specific preventive in infectious maladies. The physicians of the East make this a constant practice in the case of infectious maladies.—*Journal de Med. de Paris.*

